# Analysis of the Accuracy of Magnetic Resonance Enterography for the Detection of Active Inflammation and Disease Activity in Patients With Crohn’s Disease: A Single-Center Experience in Najran, Saudi Arabia

**DOI:** 10.7759/cureus.52674

**Published:** 2024-01-21

**Authors:** Qaed S Alhammami

**Affiliations:** 1 Radiology, Najran University, Najran, SAU

**Keywords:** crohn’s disease, cd, chronic inflammatory bowel disease, ibd, magnetic resonance enterography, mre

## Abstract

Background: Magnetic resonance enterography (MRE) is a non-invasive diagnostic imaging modality that has been used for the detection of active inflammation and disease activity in patients with Crohn's disease. However, its diagnostic accuracy in the Najran population has not been well-studied.

Study aim: This study aimed to assess the diagnostic accuracy of MRE in detecting active inflammation and disease activity in patients with Crohn's disease in the Najran population.

Methods: The study included 51 patients with Crohn's disease, and their demographic, clinical, radiological, laboratory, and endoscopic data were analyzed.

Results: The results showed that MRE findings, such as the radiological score for active inflammation and the presence of extra-intestinal manifestations, were significantly associated with the final diagnosis of active inflammation. Furthermore, the timing of MRE in relation to symptom activity and the indication for performing MRE were significantly associated with the final diagnosis. The study findings demonstrate the potential of MRE as a valuable tool for diagnosing and assessing disease activity in Crohn's disease patients in the Najran population.

## Introduction

Crohn’s disease (CD) is a chronic inflammatory bowel disease (IBD) characterized by transmural inflammation of the gastrointestinal tract, which can affect any part of the digestive system [1]. The etiology of CD is not yet fully understood, but it is believed to involve a combination of genetic, environmental, and immune factors. The symptoms of CD can vary widely, but typically include abdominal pain, diarrhea, and weight loss and can be accompanied by extra-intestinal manifestations, such as joint pain, skin rashes, and eye inflammation [1,2].

Magnetic resonance enterography (MRE) is a non-invasive imaging technique that uses magnetic resonance imaging (MRI) to produce high-resolution images of the small intestine. MRE has become increasingly popular as a diagnostic tool for CD, as it is able to provide detailed information about the presence and extent of inflammation in the intestinal wall, as well as the severity of the disease [3]. The use of MRE has several advantages over other diagnostic methods, such as computed tomography (CT) and colonoscopy, as it is non-invasive, does not involve ionizing radiation, and can be performed without the need for sedation [4].

Despite the increasing use of MRE for the diagnosis and monitoring of CD, there is still some debate about its accuracy in detecting active inflammation and disease activity, particularly in different patient populations. Previous studies have suggested that MRE may be less accurate in detecting inflammation in patients with small bowel disease, as well as in patients with structuring or penetrating disease. Additionally, the accuracy of MRE may be affected by the presence of confounding factors, such as medication use, disease duration, and previous surgical interventions [3-5]. This study aims to analyze the accuracy of MRE in detecting active inflammation and disease activity in patients with CD in the Najran population of Saudi Arabia. The study also investigated factors that may affect the accuracy of MRE, such as disease location, severity, duration, concomitant medications, and previous surgical interventions.

## Materials and methods

This study employed a retrospective observational design to analyze the diagnostic accuracy of MRE for detecting active inflammation and disease activity in patients with CD. Patient data were collected from medical records, including demographic information, clinical characteristics, MRE findings, laboratory results, endoscopic findings, diagnosis, treatment outcomes, and any adverse events related to MRE or subsequent treatments.

Study participants

The study included a total of 51 patients diagnosed with CD (based on biopsy) from the Najran population. The patients were selected based on the availability of complete medical records, including MRE results and follow-up data.

Data collection

Data collection was performed by reviewing patients' medical records, including electronic health records, radiology reports, laboratory reports, endoscopy reports, and treatment records. The collected data included demographic and clinical characteristics: age, gender, Crohn's disease diagnosis status, duration of Crohn's disease, and comorbidities; MRE findings: indication for MRE, the timing of MRE in relation to symptom activity, the radiological score for active inflammation, and presence of extra-intestinal manifestations; laboratory findings: C-reactive protein (CRP) level and erythrocyte sedimentation rate (ESR); endoscopic findings: timing of endoscopy in relation to MRE and endoscopic score for disease activity; diagnosis and treatment outcomes: final diagnosis of active inflammation or no active inflammation, treatment received (corticosteroids, immunosuppressants, biologics, surgery), and any adverse events; impact of MRE results on patient management: changes in treatment plan or initiation/discontinuation of treatment based on MRE findings; and delays in diagnosis or treatment: presence or absence of delays and reasons for delays.

Descriptive statistics were used to summarize the demographic, clinical, MRE, laboratory, and endoscopic findings. The association between variables and the final diagnosis of active inflammation or no active inflammation was assessed using chi-square tests. The level of significance was set at p < 0.05.

This study was conducted in compliance with ethical guidelines and regulations. The study protocol was reviewed and approved by the relevant ethics committee or institutional review board (Research Ethics Committee, Najran University, 202312-076-016105-03717). Patient data were anonymized and treated with confidentiality to ensure privacy and comply with data protection regulations.

## Results

The demographic and clinical characteristics of the study participants are summarized in Table 1. A total of 51 patients with CD were included in the analysis. The age distribution of the patients at the time of MRE varied, with the majority falling within the 18-30 years age group (64.7%). The age groups <18 years, 31-45 years, and 46-60 years accounted for five (9.8%), 10 (19.6%), and three (5.9%) of the patients, respectively. Regarding gender, the study population was almost evenly distributed, with 25 (49%) being female and 26 (51%) male. In terms of Crohn's disease diagnosis prior to MRE, 32 (62.7%) of the patients had a confirmed diagnosis, while 23.5% had not been diagnosed, and seven (13.7%) were uncertain about their diagnosis. The duration of CD at the time of MRE varied among the patients. Approximately nine (17.6%) of the patients had been diagnosed within the past year, while 23 (45.1%) had been living with the disease for one to five years. About seven (13.7%) had a disease duration of more than five years, and 23.5% were uncertain about the duration of their condition. Regarding comorbidities, the majority of patients 49 (96.1%) did not have any additional comorbidities. However, one patient (2%) had hypertension, and another patient (2%) had scleroderma, in addition to CD.

**Table 1:** Patient characteristics and Crohn’s disease history (n=51). N (number)

Parameter		Frequency (%)
Age, years	<18 years	5 (9.8%)
	18-30 years	33 (64.7%)
	31-45 years	10 (19.6%)
	46-60 years	3 (5.9%)
Gender	Female	25 (49%)
	Male	26 (51%)
Crohn’s disease diagnosis before MRE	Yes	32 (62.7%)
	No	12 (23.5%)
	Uncertain	7 (13.7%)
Duration of Crohn's disease at the time of MRE	<1 year	9 (17.6%)
	1-5 years	23 (45.1%)
	>5 years	7 (13.7%)
	Uncertain	12 (23.5%)
Comorbidities	None	49 (96.1%)
	Hypertension	1 (2%)
	Scleroderma	1 (2%)

Table 2 presents the MRE findings and related parameters in the study population of patients with Crohn's disease. The indication for performing MRE varied among the patients. The majority of MREs (30, 58.8%) were performed due to the presence of active symptoms. Additionally, 20 (39.2%) of the MREs were conducted for surveillance purposes, while only one (2%) of the patients had MRE performed to investigate the possibility of Crohn's disease (Query CD). It is noteworthy that MRE was performed during a period of active symptoms in 29 (56.9%) of the cases. However, for 13 (25.5%) of the patients, MRE was conducted when symptoms were not active, and nine (17.6%) had an uncertain status regarding symptom activity during the MRE procedure. The radiological score for active inflammation, as determined by MRE findings, varied among the patients. The most common score was 1, observed in 21 (41.2%) of the cases, followed by a score of 0 in 14 (27.5%) of the patients. Scores of 2 and 3 were less frequent, accounting for 12 (23.5%) and four (7.8%) of the cases, respectively. In terms of extra-intestinal manifestations detected during MRE, 15 (29.4%) of the patients had such findings, while the remaining 36 (70.6%) did not exhibit any extra-intestinal manifestations during the MRE examination.

**Table 2:** Laboratory and endoscopic findings (n=51). N (number)

Parameter		Frequency (%)
C-reactive protein (CRP) level	<5 mg/L	11 (21.6%)
	5-10 mg/L	7 (13.7%)
	>10 mg/L	9 (17.6%)
	N/A	24 (47.1%)
Erythrocyte sedimentation rate (ESR)	<20 mm/h	12 (23.5%)
	20-40 mm/h	10 (19.6%)
	>40 mm/h	6 (11.8%)
	N/A	23 (45.1%)
Endoscopy performed within 30 days of the MRE	Yes	37 (72.5%)
	No	14 (27.5%)
Endoscopic score for disease activity	0	14 (27.5%)
	1	23 (45.1%)
	2	8 (15.7%)
	3	6 (11.8%)

Table 3 outlines key findings related to laboratory and endoscopic results. The CRP levels were measured, with 21.6% having a CRP level below 5 mg/L, 13.7% ranging from 5 to 10 mg/L, and 17.6% exceeding 10 mg/L. Additionally, 47.1% had a missing or unavailable CRP level. The ESR was also evaluated, with 23.5% below 20 mm/h, 19.6% between 20 and 40 mm/h, and 11.8% above 40 mm/h, and 45.1% had an unavailable ESR value. Endoscopy was performed in 72.5% of cases within 30 days of another procedure, and the endoscopic score for disease activity varied, with 27.5% showing no disease activity, 45.1% having a score of 1, and the rest having higher scores.

**Table 3:** Diagnosis and treatment following MRE (n=51). N (number)

Parameter		Frequency (%)
Final diagnosis after clinical assessment, laboratory tests, and endoscopic findings	Active inflammation	34 (66.7%)
	No active inflammation	17 (33.5%)
Treatment after the MRE	None	5 (9.8%)
	Corticosteroids	8 (15.7%)
	Immunosuppressants	33 (64.7%)
	Biologics	3 (5.9%)
	Surgery	2 (3.9%)
Patient experience any adverse events related to the MRE or treatment	No	51 (100%)
MRE results change in patient management	No change	3 (5.9%)
	Initiation of treatment	16 (31.4%)
	Change in treatment regimen	29 (56.9%)
	Discontinuation of treatment	3 (5.9%)
Delay in diagnosis or treatment of Crohn's disease	Yes	9 (17.6%)
	No	42 (82.4%)
Reason for the delay	No delay	42 (82.4%)
	Delay in interpreting MRE	6 (11.8%)
	Delay in performing endoscopy	2 (3.9%)
	Delay in interpreting endoscopy	1 (2%)

Table 4 presents the diagnosis and treatment outcomes after conducting MRE in the study population of patients with CD. The most common indication for performing MRE was active symptoms, and MRE was often conducted during a period of active symptoms. The radiological score for active inflammation varied, with a significant number of patients having a score of 1. Some patients had extra-intestinal manifestations detected during MRE. Laboratory findings showed varied levels of CRP and ESR, with a considerable portion of patients having unavailable values. Endoscopy was performed in most cases, and the endoscopic score for disease activity varied. Following MRE, most patients were diagnosed with active inflammation, and treatment often involved immunosuppressants. No adverse events were reported, and MRE results led to changes in patient management, including treatment initiation and regimen adjustments. Some patients experienced delays in the diagnosis or treatment of Crohn's disease, often due to delays in interpreting MRE results or performing endoscopy.

**Table 4:** Final diagnosis in association with patient and disease characteristics (n=51). A p-value of less than or equal to 0.05 is regarded as evidence of a statistically significant results.

Parameter		Final diagnosis		X^2^	P-value
		Active inflammation	No active inflammation		
Age, years	<18 years	4 (11.8%)	1 (5.9%)	11.8	0.008
	18-30 years	26 (76.5%)	7 (41.2%)		
	31-45 years	4 (11.8%)	6 (35.3%)		
	46-60 years	0 (0%)	3 (17.6%)		
Gender	Female	19 (55.9%)	6 (35.3%)	1.9	0.166
	Male	15 (44.1%)	11 (64.7%)		
Crohn’s disease diagnosis before MRE	Yes	20 (58.8%)	12 (70.6%)	1.4	0.498
	No	8 (23.5%)	4 (23.5%)		
	Uncertain	6 (17.6%)	1 (5.9%)		
Duration of Crohn's disease at the time of MRE	<1 year	7 (20.6%)	2 (11.8%)	5.5	0.137
	1-5 years	16 (47.1%)	7 (41.2%)		
	>5 years	2 (5.9%)	5 (29.4%)		
	Uncertain	9 (26.5%)	3 (17.6%)		
Comorbidities	None	34 (100%)	15 (88.2%)	4.2	0.125
	Hypertension	0 (0%)	1 (5.9%)		
	Scleroderma	0 (0%)	1 (5.9%)		

Table 5 provides an analysis of the relationship between patient and disease characteristics and the final diagnosis of active inflammation or no active inflammation in patients with Crohn's disease. Age was found to be significantly associated with the final diagnosis. Among patients aged less than 18 years, two (11.8%) had active inflammation, while only one (5.9%) had no active inflammation. In the age group of 18-30 years, the majority 26 (76.5%) had active inflammation, compared to seven (41.2%) who had no active inflammation. For the age groups of 31-45 years and 46-60 years, two (11.8%) and three (17.6%) had active inflammation, respectively, while six (35.3%) and six (17.6%) had no active inflammation. These findings indicate a significant association between age and the presence of active inflammation (χ2=11.8, p-value=0.008). Gender was also examined in relation to the final diagnosis, although the association did not reach statistical significance. Among females, 19 (55.9%) were diagnosed with active inflammation, while six (35.3%) had no active inflammation. Among males, 15 (44.1%) had active inflammation, and 11 (64.7%) had no active inflammation. The chi-square test showed a non-significant association between gender and the final diagnosis (χ2=1.9, p-value=0.166). The timing of Crohn's disease diagnosis before MRE did not demonstrate a significant association with the final diagnosis. Among patients with a previous diagnosis, 20 (58.8%) had active inflammation, while 12 (70.6%) of those without a previous diagnosis had active inflammation. Patients with uncertain Crohn's disease diagnosis showed a relatively equal distribution between active and no active inflammation. The chi-square test indicated a non-significant association between the timing of diagnosis and the final diagnosis (χ2=1.4, p-value=0.498). The duration of Crohn's disease at the time of MRE also did not exhibit a significant association with the final diagnosis. For patients with a duration of less than one year, 20.6% had active inflammation, while two (11.8%) had no active inflammation. Among those with a duration of one to five years, 16 (47.1%) had active inflammation, and seven (41.2%) had no active inflammation. Patients with a duration of more than five years displayed 5.9% with active inflammation and five (29.4%) with no active inflammation. The chi-square test yielded a non-significant association between the duration of Crohn's disease and the final diagnosis (χ2=5.5, p-value=0.137). Considering comorbidities, no significant association was observed between the presence of comorbidities and the final diagnosis. Among patients without comorbidities, 100% had active inflammation, while 15 (88.2%) of those with comorbidities had active inflammation. However, the chi-square test did not show a significant association between comorbidities and the final diagnosis (χ2=4.2, p-value=0.125).

**Table 5:** Final diagnosis in association with MRE findings (n=51). A p-value of less than or equal to 0.05 is regarded as evidence of a statistically significant results.

Parameter		Final diagnosis		X^2^	P-value
		Active inflammation	No active inflammation		
Indication for performing MRE	Active symptoms	27 (79.4%)	3 (17.6%)	18.4	0.000
	Surveillance	7 (20.6%)	13 (76.5%)		
	Query CD	0 (0%)	1 (5.9%)		
MRE performed during a period of active symptoms	Yes	28 (82.4%)	1 (5.9%)	27.3	0.000
	No	3 (8.8%)	10 (58.8%)		
	Uncertain	3 (8.8%)	6 (35.3%)		
Radiological score for active inflammation	0	0 (0%)	14 (82.4%)	39.4	0.000
	1	18 (52.9%)	3 (17.6%)		
	2	12 (35.3%)	0 (0%)		
	3	4 (11.8%)	0 (0%)		
Extra-intestinal manifestations detected during MRE	Yes	14 (41.2%)	1 (5.9%)	6.8	0.009
	No	20 (58.8%)	16 (94.1%)		

Table 6 presents the relationship between MRE findings and the final diagnosis of active inflammation or no active inflammation in patients with Crohn's disease. The indication for performing MRE was found to have a significant association with the final diagnosis. Among patients with active symptoms as the indication for MRE, a majority 27 (79.4%) were diagnosed with active inflammation, while only 17.6% had no active inflammation. In contrast, for patients undergoing MRE for surveillance purposes, the majority 13 (76.5%) had no active inflammation, with only seven (20.6%) showing active inflammation. The association between the indication for MRE and the final diagnosis was statistically significant (χ2=18.4, p-value=0). Furthermore, performing MRE during a period of active symptoms demonstrated a strong association with the final diagnosis. Patients who had MRE during a period of active symptoms were mostly diagnosed with active inflammation (82.4%), while only 5.9% had no active inflammation. On the other hand, patients who did not have active symptoms during MRE had a higher proportion of no active inflammation (10, 58.8%) compared to active inflammation (3, 8.8%). The association between MRE timing and the final diagnosis was highly significant (χ2=27.3, p-value=0). The radiological score for active inflammation showed a significant association with the final diagnosis. Among patients with a radiological score of 0, none had active inflammation, while the majority 14 (82.4%) had no active inflammation. In contrast, as the radiological score increased, there was a corresponding increase in the proportion of patients with active inflammation. For a score of 1, 18 (52.9%) had active inflammation, and for scores of 2 and 3, the proportions were six (35.3%) and four (11.8%), respectively. The association between the radiological score and the final diagnosis was highly significant (χ2=39.4, p-value=0). Extra-intestinal manifestations detected during MRE also showed a significant association with the final diagnosis. Patients with extra-intestinal manifestations had a higher likelihood of being diagnosed with active inflammation (14, 41.2%) compared to those without such manifestations (one, 5.9%). The association between the presence of extra-intestinal manifestations and the final diagnosis was statistically significant (χ2=6.8, p-value=0.009).

**Table 6:** Final diagnosis in association with laboratory and endoscopic findings (n=51). A p-value of less than or equal to 0.05 is regarded as evidence of a statistically significant results.

Parameter		Final diagnosis		X^2^	P-value
		Active inflammation	No active inflammation		
C-reactive protein (CRP) level	<5 mg/L	2 (5.9%)	9 (52.9%)	15.4	0.002
	5-10 mg/L	5 (14.7%)	2 (11.8%)		
	>10 mg/L	8 (23.5%)	1 (5.9%)		
	N/A	19 (55.9%)	5 (29.4%)		
Erythrocyte sedimentation rate (ESR)	<20 mm/h	2 (5.9%)	10 (58.8%)	18.7	0.000
	20-40 mm/h	8 (23.5%)	2 (11.8%)		
	>40 mm/h	6 (17.6%)	0 (0%)		
	N/A	18 (52.9%)	5 (29.4%)		
Endoscopy performed within 30 days of the MRE	Yes	25 (73.5%)	12 (70.6%)	0.05	0.824
	No	9 (26.5%)	5 (29.4%)		
Endoscopic score for disease activity	0	1 (2.9%)	13 (76.5%)	31.9	0.000
	1	19 (55.9%)	4 (23.5%)		
	2	8 (23.5%)	0 (0%)		
	3	6 (17.6%)	0 (0%)		

Table 7 explores the relationship between laboratory and endoscopic findings and the final diagnosis of active inflammation or no active inflammation in patients with Crohn's disease. The C-reactive protein (CRP) level exhibited a significant association with the final diagnosis. Patients with a CRP level less than 5 mg/L had a higher proportion of no active inflammation (nine, 52.9%) compared to active inflammation (one, 5.9%). Conversely, patients with CRP levels greater than 10 mg/L had a higher proportion of active inflammation (eight, 23.5%) compared to no active inflammation (one, 5.9%). The association between CRP levels and the final diagnosis was statistically significant (χ2=15.4, p-value=0.002). Similarly, the ESR demonstrated a significant association with the final diagnosis. Patients with an ESR of less than 20 mm/h had a higher proportion of no active inflammation (10, 58.8%) compared to active inflammation (one, 5.9%). On the other hand, patients with an ESR greater than 40 mm/h had a higher proportion of active inflammation (six, 17.6%), and no patients in this category had no active inflammation. The association between ESR and the final diagnosis was highly significant (χ2=18.7, p-value=0). The timing of endoscopy, whether performed within 30 days of MRE or not, did not show a significant association with the final diagnosis. There was no substantial difference in the proportions of patients with active or no active inflammation based on the timing of endoscopy (χ2=0.05, p-value=0.824). However, the endoscopic score for disease activity displayed a strong association with the final diagnosis. Patients with an endoscopic score of 0 had a significantly higher proportion of no active inflammation (13, 76.5%) compared to active inflammation (one, 2.9%). In contrast, patients with scores of 1, 2, and 3 had higher proportions of active inflammation compared to no active inflammation. The association between the endoscopic score and the final diagnosis was highly significant (χ2=31.9, p-value=0).

**Table 7:** Final diagnosis in association with laboratory and endoscopic findings (n=51). A p-value of less than or equal to 0.05 is regarded as evidence of statistically significant results.

Parameter		Final diagnosis		X^2^	P-value
		Active inflammation	No active inflammation		
C-reactive protein (CRP) level	<5 mg/L	2 (5.9%)	9 (52.9%)	15.4	0.002
	5-10 mg/L	5 (14.7%)	2 (11.8%)		
	>10 mg/L	8 (23.5%)	1 (5.9%)		
	N/A	19 (55.9%)	5 (29.4%)		
Erythrocyte sedimentation rate (ESR)	<20 mm/h	2 (5.9%)	10 (58.8%)	18.7	0.000
	20-40 mm/h	8 (23.5%)	2 (11.8%)		
	>40 mm/h	6 (17.6%)	0 (0%)		
	N/A	18 (52.9%)	5 (29.4%)		
Endoscopy performed within 30 days of the MRE	Yes	25 (73.5%)	12 (70.6%)	0.05	0.824
	No	9 (26.5%)	5 (29.4%)		
Endoscopic score for disease activity	0	1 (2.9%)	13 (76.5%)	31.9	0.000
	1	19 (55.9%)	4 (23.5%)		
	2	8 (23.5%)	0 (0%)		
	3	6 (17.6%)	0 (0%)		

**Figure 1:**
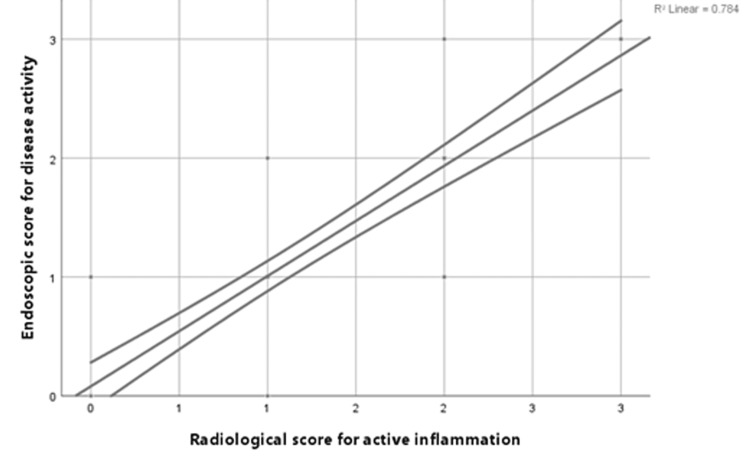
Simple scatter plot with fit live of the endoscopic score for disease activity by the radiological score for active inflammation. Pearson correlation=0.885, p=0.000

Figure 1 presents the correlation between the radiological score of active inflammation and the endoscopic score for disease activity in patients with Crohn's disease. The figure indicates a strong positive correlation between these two variables, as evidenced by the Pearson correlation coefficient of 0.885 (p-value=0.000). The upward trend in the scatterplot suggests that, as the endoscopic score for disease activity increases, the radiological score of active inflammation also tends to increase. This positive correlation implies that higher endoscopic scores, indicating more severe disease activity, are associated with higher radiological scores, indicating a greater degree of active inflammation observed through radiological imaging techniques. The significant p-value (p=0.000) further confirms the strength of the correlation observed in the data.

## Discussion

MRE is a non-invasive imaging technique that uses magnetic fields and radio waves to create detailed images of the small bowel and colon. MRE has several advantages over other imaging modalities such as computed tomography (CT) enterography and small bowel follow-through (SBFT), including better soft tissue contrast, lack of ionizing radiation, and improved visualization of the bowel wall [6-10]. Studies have evaluated the diagnostic accuracy of MRE for detecting active inflammation and disease activity in patients with Crohn's disease. A meta-analysis found that MRE has a high specificity of 95% (95% CI: 0.92-0.97) to detect colon pathologies in CD, while the sensitivity is low at 69% (95% CI: 0.52-0.82) [11].

The aim of this study was to analyze the accuracy of MRE in detecting active inflammation and disease activity in patients with Crohn's disease in the Najran population. The study population consisted of 51 patients with Crohn's disease in the Najran population. Most patients had a confirmed diagnosis of Crohn's disease prior to MRE, while some were either undiagnosed or uncertain about their diagnosis. The duration of Crohn's disease varied among the patients. The MRE findings indicated that the majority of MREs were performed due to the presence of active symptoms. The radiological score for active inflammation varied among the patients, with the most common score being 1 [12]. Extra-intestinal manifestations were detected in a percentage of the patients.

The MRE findings in this study, including the radiological score for active inflammation, indicate a range of disease severity, with the most common score being 1, followed by scores of 0, 2, and 3. These findings align with previous studies that have used MRE to assess disease activity in Crohn's disease patients [12-14].

The detection of extra-intestinal manifestations during MRE in 29.4% of the patients is consistent with the known association between Crohn's disease and various extra-intestinal manifestations, such as joint inflammation, skin lesions, and ocular involvement. The presence of these manifestations may have implications for disease management and treatment decisions [15].

Laboratory findings, such as CRP levels and ESR, were evaluated in relation to the final diagnosis. The study found a significant association between higher CRP levels and the presence of active inflammation. Similarly, higher ESR levels were associated with active inflammation. Endoscopy was performed in the majority of cases within 30 days of MRE, and the endoscopic score for disease activity showed varying degrees of inflammation.

The final diagnosis revealed that a majority of the participants were diagnosed with active inflammation, indicating disease activity. The remaining participants had no active inflammation detected. In terms of treatment outcomes, a variety of approaches were implemented based on the MRE results, including the prescription of corticosteroids, immunosuppressants, and biologics, as well as surgical interventions. No adverse events related to MRE or subsequent treatment were reported by the patients, indicating the safety of the procedures.

The use of endoscopy as a reference standard in this study is consistent with the current clinical practice for evaluating disease activity in Crohn's disease. The distribution of endoscopic scores reflects varying levels of disease activity, with higher scores indicating more severe inflammation [16].

The impact of MRE results on patient management was assessed, and the study found that a significant percentage of patients experienced changes in their treatment regimen based on MRE findings. Furthermore, delays in the diagnosis or treatment of Crohn's disease were observed in a minority of patients, with the most common reason being delays in the interpretation of MRE results.

A multicentre trial aimed to determine the comparative accuracy of MRE and ultrasound for imaging Crohn's disease among patients with newly diagnosed or established Crohn's disease found that the sensitivity of MRE for small bowel disease extent and presence was found to be significantly greater than that of ultrasound, with a 10% difference for extent and a 5% difference for presence. The specificity of MRE for small bowel disease extent was also significantly greater than that of ultrasound, with a difference of 14%. Both MRE and ultrasound were found to have high sensitivity for detecting small bowel disease presence and were viable alternatives to ileocolonoscopy. However, MRE was generally preferred in a national health service setting due to its superior sensitivity and specificity compared to ultrasound [17].

## Conclusions

In conclusion, this study provides valuable insights into the accuracy of MRE in detecting active inflammation and disease activity in patients with Crohn's disease in the Najran population. The findings support the use of MRE as a valuable diagnostic tool, with significant associations found between MRE findings, laboratory parameters, and the final diagnosis. The study highlights the impact of MRE results on patient management and emphasizes the importance of timely interpretation of MRE findings to minimize delays in diagnosis or treatment. We recommend that future studies should involve larger multicenter cohorts to enhance the generalizability and robustness of the findings.
